# A second‐generation capture panel for cost‐effective sequencing of genome regulatory regions in wheat and relatives

**DOI:** 10.1002/tpg2.20296

**Published:** 2022-12-09

**Authors:** Junli Zhang, Juan M. Debernardi, Germán F. Burguener, Frédéric Choulet, Etienne Paux, Lauren O'Connor, Jacob Enk, Jorge Dubcovsky

**Affiliations:** ^1^ Dep. of Plant Sciences Univ. of California Davis CA 95616 USA; ^2^ UCA, INRAE, GDEC Clermont‐Ferrand 63000 France; ^3^ VetAgro Sup formerly INRAE Lempdes France; ^4^ Daicel Arbor Biosciences Ann Arbor MI 48103 USA; ^5^ Howard Hughes Medical Institute Chevy Chase MD 20815 USA

## Abstract

As genome resources for wheat (*Triticum* L.) expand at a rapid pace, it is important to update targeted sequencing tools to incorporate improved sequence assemblies and regions of previously unknown significance. Here, we developed an updated regulatory region enrichment capture for wheat and other *Triticeae* species. The core target space includes sequences from 2‐Kbp upstream of each gene predicted in the Chinese Spring wheat genome (IWGSC RefSeq Annotation v1.0) and regions of open chromatin identified with an assay for transposase‐accessible chromatin using sequencing from wheat leaf and root samples. To improve specificity, we aggressively filtered candidate repetitive sequences using a combination of nucleotide basic local alignment search tool (BLASTN) searches to the Triticeae Repetitive Sequence Database (TREP), identification of regions with read over‐coverage from previous target enrichment experiments, and k‐mer frequency analyses. The final design comprises 216.5 Mbp of predicted hybridization space in hexaploid wheat and showed increased specificity and coverage of targeted sequences relative to previous protocols. Test captures on hexaploid and tetraploid wheat and other diploid cereals show that the assay has broad potential utility for cost‐effective promoter and open chromatin resequencing and general‐purpose genotyping of various *Triticeae* species.

## Core Ideas

- Regulatory regions contain variation that is valuable for crop improvement.
- Promoter regions and open chromatin regions are enriched in regulatory elements.
- A new target enrichment assay facilitates cost‐effective regulatory region sequencing in wheat and relatives.
- The new assay improves in scope and performance by incorporating new target space and avoiding repetitive elements.

**Abbreviations**
ATAC‐seq: Assay for Transposase‐Accessible Chromatin using sequencing
BED: Browser Extensible Data
BLASTN: nucleotide basic local alignment search tool
CS: Chinese Spring
EMS: ethyl methanesulfonate
MAPS: mutations and polymorphisms surveyor
PCR: polymerase chain reaction
SNP: single nucleotide polymorphism

## 1 INTRODUCTION

The coordinated action of promoters and enhancers is critical to regulate the precise spatial and temporal patterns of gene expression required for organismal development and appropriate responses to environmental changes (Wittkopp & Kalay, 2012). Sequence‐level variation in regulatory elements is a major driver of phenotypic variation and adaptation (Rodgers‐Melnick et al., 2016) and, therefore, genomic tools are necessary to access this variability. This is particularly relevant in crop species, where variation in regulatory regions can be used to improve economically valuable traits. Variants in regulatory regions are especially useful for plant improvement projects because they usually cause less severe phenotypic changes than gene knockout mutations. Regulatory mutations are expected to affect the timing, spatial distribution or levels of gene expression rather than the function of all the transcripts (Rodriguez‐Leal et al., 2017; Wittkopp & Kalay, 2012).

In species with small genomes, such as *Arabidopsis* and rice (<1 Gbp), natural variation in regulatory and coding regions can be cost‐effectively accessed by whole genome resequencing of a large number of accessions. However, this strategy is not economically viable in polyploid species such as wheat (*Triticum* L.), that have multiple copies of 4‐to‐5‐Gb‐sized subgenomes composed mainly of repetitive elements (International Wheat Genome Sequencing Consortium, 2018). Instead, capture platforms have been used to sequence the coding and regulatory regions of the wheat genome. A variety of exome‐targeting probe sets for wheat have been designed using various commercial platforms resulting in useful studies (Chen et al., 2021; Dang et al., 2022; Gabay et al., 2021; Glenn et al., 2022; Krasileva et al., 2017; Serra et al., 2021). However, some of those capture assays have been discontinued and would be expensive to reproduce as new custom assays for small or moderate sample numbers. Currently, only the Wheat Exome version 1 design offered by Daicel Arbor Biosciences (hereafter “Arbor”) is available as an off‐the‐shelf wheat probe set.

A global wheat promoter capture assay was developed after the release of the Chinese Spring RefSeq v1.0 sequence (International Wheat Genome Sequencing Consortium, 2018) covering 2 Kbp of sequence upstream of the start codon of all high‐confidence annotated genes (Gardiner et al., 2019). However, this design is among the ones that were recently removed from public availability. In this study, we present a new improved and expanded regulatory capture design developed in collaboration with Arbor, which they offer as a catalog product for broad community use. Our new design leverages data obtained from capture experiments using the previous promoter probe design and eliminates previously undetected repetitive regions. It also expands the scope of regulatory targets by including open chromatin regions identified with an assay for transposase‐accessible chromatin using sequencing (ATAC‐seq; Buenrostro et al., 2013) data from wheat leaf protoplasts (Lu et al., 2020) and roots (Debernardi et al., 2022). Finally, the behavior of the probe set for enrichment efficiency and overall genomic coverage depth and breadth is evaluated in several *Triticum*, *Aegilops*, and *Secale* samples.

**Core Ideas**

- Regulatory regions contain variation that is valuable for crop improvement.
- Promoter regions and open chromatin regions are enriched in regulatory elements.
- A new target enrichment assay facilitates cost‐effective regulatory region sequencing in wheat and relatives.
- The new assay improves in scope and performance by incorporating new target space and avoiding repetitive elements.

## 2 MATERIALS AND METHODS

### 2.1 Capture design: promoter sequences

Target design began with the same 2‐Kbp regions upstream of the 110,790 high‐confidence wheat genes annotated in Chinese Spring (CS) RefSeq v1.0 that were used in the previous promoter capture design (Gardiner et al., 2019), henceforth ‘Gardiner probe set.’ This initial sequence included 110,790 distinct regions covering a total of 221.6 Mbp. We then excluded all the repetitive elements annotated in the wheat genome annotation RefSeq v1.1 (International Wheat Genome Sequencing Consortium, 2018). The subtraction of the corresponding browser extensible data (BED) files yielded a reduced sequence space of 168.8 Mbp (23.6% reduction).

We complemented this set of putative, single‐copy hexaploid promoter regions with sequences from the tetraploid wheat Kronos [PI 576168, *Triticum turgidum* L. subsp. *durum* (Desf.) Husn.]. These sequences were derived from 40,975 contigs of Kronos (33.4 Mbp) assembled from Kronos reads that did not map to CS (Krasileva et al., 2017). We then aligned these contigs with the CS promoter space using nucleotide basic local alignment search tool (BLASTN; less stringent than read mapping) and selected 13.5 Mbp of putative promoter regions, bringing the working target space to 182.4 Mbp. To further reduce sequence redundancy, we clustered the collection of contiguous sequences using a threshold of 99% identity with the software CD‐HIT‐EST v4.7 (Fu et al., 2012), which resulted in 4.3% reduction of the sequence space to 174.6 Mbp. We then eliminated contiguous sequences smaller than 100 bp, bringing the final starting space for probe design to 174.0 Mbp represented by 167,685 contiguous sequences.

To eliminate repetitive sequences missed in the RefSeq v1.1 annotation, we performed additional filtering steps. First, we used BLASTN to query our 167,685 target sequences against the database of Triticeae Repetitive elements (TREP; Wicker et al., 2002) and removed significant similar sequences (E < 1e^−10^). Then, we performed a k‐mer analysis using Tallymer v1.6.1 (Kurtz et al., 2008) and an index previously created based on Chinese Spring RefSeq v1.0 (k‐mer length = 17) (International Wheat Genome Sequencing Consortium, 2018). We evaluated four different thresholds of k‐mer occurrence: 5, 10, 50, and 100, which masked 55, 36, 13, and 8% of the regulatory regions, respectively. To avoid masking conserved regulatory regions, we decided to mask 17‐mers repeated 100 times or more in the genome. Finally, we analyzed 20 captures performed in tetraploid Kronos with the Gardiner probe set, and masked regions with a coverage higher than 100×, which is more than five‐fold higher than the average coverage of these captures. The additional masking generated new fragmentation, so we removed one last time sequences of contiguous length lower than 100 nt. The final targeted putative promoter space was 162.5 Mbp.

### 2.2 Capture design: open chromatin regions from ATAC‐seq

To include additional regulatory sequences, we added open chromatin regions from publicly available wheat ATAC‐seq data from wheat leaf protoplasts (Lu et al., 2020) and from seminal roots recently generated in our lab (Debernardi et al., 2022). Root ATAC‐seq data was generated from the tetraploid cultivar Kronos, whereas leaf ATAC‐seq data was generated from leaf protoplast isolated from the hexaploid cultivar Paragon. Raw data from both studies was analyzed using MACS2 with the same parameters (Debernardi et al., 2022).

The initial ATAC‐seq peaks covered 28.36 Mbp (57,981 peaks) in the leaf protoplast data and 2.75 Mb in the frozen root data (7,269 peaks). Among these peaks, we identified 4,432 (61%) that overlapped in at least 10% of their length, suggesting a substantial proportion of shared open chromatin regions. Excluding the 1.53 Mbp of overlapping sequences, we identified 29.58 Mbp of non‐redundant ATAC‐seq data between the two datasets. From this we subtracted the 6.1 Mbp already present in the promoter sequences and added 23.5 Mbp of new sequences to the regulatory target design.

### 2.3 Probe design

Each selected target sequence from the promoters and ATAC‐seq was padded by 100 nt on either side and remerged, comprising 241.59 Mbp of potential hybridization target. We used an “island” approach for tiling probes, wherein we selected 80‐nt probes with the best predicted hybridization dynamics in every 100‐nt window across the padded space. These probe candidates were then aggressively filtered for specificity against both RefSeq v1.0 and the draft Kronos assembly in order to once again minimize the likelihood of targeting multicopy sequence motifs. To describe the final hybridization target space on RefSeq 1.0, we mapped the probes to the reference genome using *bwa mem* (version 0.7.10‐r789, default parameters), and padded these map sites by 100 nucleotides to reflect a likely retrievable space of roughly 216.47 Mbp (DAB_WheatRegulatoryV1.IWGSCv1_hybspace.bed), which we refer to hereafter as ‘hybridization space’. Roughly 168.0 Mbp of this space intersects with the original non‐padded target space on hexaploid RefSeq v1.0, which we refer to as the final ‘target space’ (DAB_WheatRegulatoryV1.IWGSCv1.bed). To determine the corresponding regions on the rye assembly, we used BLAST blastn (version 2.6.0+) to map the target space wheat sequences to the rye genome, after which the top bit score intervals per query sequence were retained and merged (DAB_WheatRegulatoryV1.Weiningv1.bed and DAB_WheatRegulatoryV1.Weiningv1_hybspace.bed, respectively).

The probe set was synthesized in subgenome‐specific modules, so that a user can exclude probes for a subgenome not present in the sample being enriched, or otherwise customize the composition of enrichment reactions. Supplemental Table S1 summarizes the total target and hybridization spaces for each of the reference genomes and sub‐genomes (for the polyploid species), as well as the names of the BED files deposited in Zenodo (Zhang et al., 2022).

### 2.4 Capture performance evaluation

Test materials included genomic DNAs extracted from eight hexaploid accessions (*T. aestivum*, genomes ABD), 24 tetraploid Kronos ethyl methanesulfonate (EMS) mutants [*T. turgidum* subsp. *durum*, genomes AB] (Krasileva et al., 2017), and 16 diploid accessions. The diploid accessions included eight from *T. monococcum* (A^m^ genome), two from *T. urartu* (A genome), three from *Aegilops speltoides* (S genome), one from *Aegilops markgrafii* (C genome) and two from *Secale cereale* (R genome; Supplemental Table S2).

To generate Illumina libraries, genomic DNA was sonicated with a Q800R instrument (Qsonica) to mean lengths of 400 bp and purified with dual‐sided Solid Phase Reversible Immobilization (SPRI) treatment (Beckman Coulter Life Sciences). Then either 200‐ (hexaploid and tetraploids) or 100‐ng (diploids) sonicated and size‐selected gDNA was taken to end repair, A‐tailing, and adapter ligation using the KAPA HyperPrep DNA kit (Roche). Each ligation product was index‐amplified using unique dual 8‐bp indexing primers for eight cycles with KAPA HiFi polymerase (Roche).

For hexaploid *T. aestivum*, Kronos‐specific probes were excluded from the captures. For tetraploid *T. turgidum* ssp. *durum* captures, the D‐specific probes were excluded. For diploid wheat and rye, we captured several taxa in the same pools, so we included all probe modules. For the captures, we pooled eight libraries in hexaploid wheat (1 μg each), 12 in tetraploid wheat (750 ng each) and 16 in the diploid species (500 ng each). Captures were conducted following the protocol described in myBaits Expert Wheat Exome kit (Arbor Biosciences, 2021).

We sequenced the resulting capture pools on an Illumina NovaSeq 6000 S4 flow‐cell using a PE150 protocol, with sample demultiplexing requiring 100% match to both the i5 and i7 indexes. To analyze target enrichment specificity, we down‐sampled the reads to 1‐M read‐pairs (2‐M reads) prior to reference mapping. For analysis of coverage depth and breadth and for variant calling, we down‐sampled to 60‐M pairs (18 Gbp) for the hexaploid accessions, 40‐M pairs (12 Gbp) for the tetraploid accessions, and 20‐M pairs (6 Gbp) for the diploid species, which follows recommended sequencing depths for analysis of data generated with Arbor's Wheat Exome V1 kit. Some samples did not yield this minimum number of reads and so were excluded from coverage analysis (Supplemental Table S2). After down‐sampling, reads were taken directly to reference alignment with *bwa mem* (version 0.7.10‐r789, default parameters) to either taxon‐appropriate subgenome sets of Chinese Spring RefSeq v1.0 (International Wheat Genome Sequencing Consortium, 2018), or to the genome assembly of Weining rye (Li et al., 2021). In both cases, mapping was performed to “parts” versions of each genome assembly, wherein each chromosome was divided into two (wheat) or three (rye) parts to make them compatible with *bwa* reference indexing. Following polymerase chain reaction (PCR) duplicate removal with *picard MarkDuplicates* (version 2.18.15‐SNAPSHOT), coverage depth and breadth were assessed with *bedtools* (version 2.17.0), and variants were called using *bcftools* (version 1.10.2‐105‐g7cd83b7, default parameters) with a minimum quality score of 20 and read depth of 10. Building information modelling (BIM) collaboration format (BCF) files with the variant calls were also deposited in Zenodo (Zhang et al., 2022).

### 2.5 Comparison of this and previous promoter capture assays in tetraploid wheat

To compare the behavior of this new capture probe set and protocol to those described by Gardiner et al. (2019), we enriched libraries built from 24 Kronos EMS‐mutagenized lines included in a previous exome capture study (Krasileva et al., 2017). DNA extraction, construction of the sequencing libraries, and capture followed procedures described previously (Krasileva et al., 2017). Target enrichment was performed in the same set of 24 tetraploid samples using both the promoters‐only probes in the Gardiner probe set, ordered as a custom kit from Roche (SeqCap EZ Prime Developer Probes, cat# 8247633001), and the Arbor assay described in this study.

Each enrichment reaction was performed on a pool of 12 libraries (125 ng per library to be consistent with previous protocols). Following enrichment, the captured DNA was amplified for 10 cycles using KAPA HiFi HotStart ReadyMix (6.25 ml, Roche, catalog No. 7958935001) and purified in 1.8x volume of Agencourt AMPure beads (Beckman Coulter, catalog No. A63881). Captured DNA was eluted in 30 μl of ultrapure water and quantified using QUBIT 2.0. Following enrichment, the pools were sequenced on one lane of Illumina NovaSeq S4 (PE150) at the Genome Center of University of California‐Davis, and informatically processed in identical fashion as the main capture tests.

After sequencing, we used a similar analysis procedure as was used for general capture metrics in our test set. First, read data was down‐sampled to the same level as before (40‐M pairs). Then we estimated the target region coverage depth (as defined by the subgenome A, B, and Un subgenome entries of “Prom‐capture‐HC+5UTR‐targets.bed” from Gardiner et al., 2019), the percent of reads that mapped to unique locations, the percent of reads on‐target, and the percent of duplicated reads to gauge overall library complexity at this raw read depth. We also compared the number of EMS mutations detected with the two capture protocols using the mutations and polymorphisms surveyor (MAPS) pipeline described previously (Henry et al., 2014). This pipeline eliminates polymorphisms between Kronos and CS and potential homoeologous polymorphism by excluding duplicated single nucleotide polymorphisms (SNPs) in sets of 24 libraries analyzed simultaneously (Henry et al., 2014; Krasileva et al., 2017). To avoid sequencing errors, we only called mutations that were detected at least four times for heterozygous mutations and at least three times for homozygous mutations. In the previous exome capture, this threshold resulted in an error rate lower than 0.3% (Krasileva et al., 2017).

## 3 RESULTS

### 3.1 Promoter capture and ATAC‐seq

The combined 2‐kb promoter regions in front of all the high confidence annotated genes resulted in an initial 221.6‐Mbp sequence space. However, after the multiple filtering steps for repetitive sequences described in the Materials and Methods section, this space was reduced to 162.5 Mbp (26.7% reduction). These promoter sequences were then complemented with open chromatin sequences obtained from roots and leaf protoplasts ATAC‐seq data (Debernardi et al., 2022; Lu et al., 2020).

**FIGURE 1:**
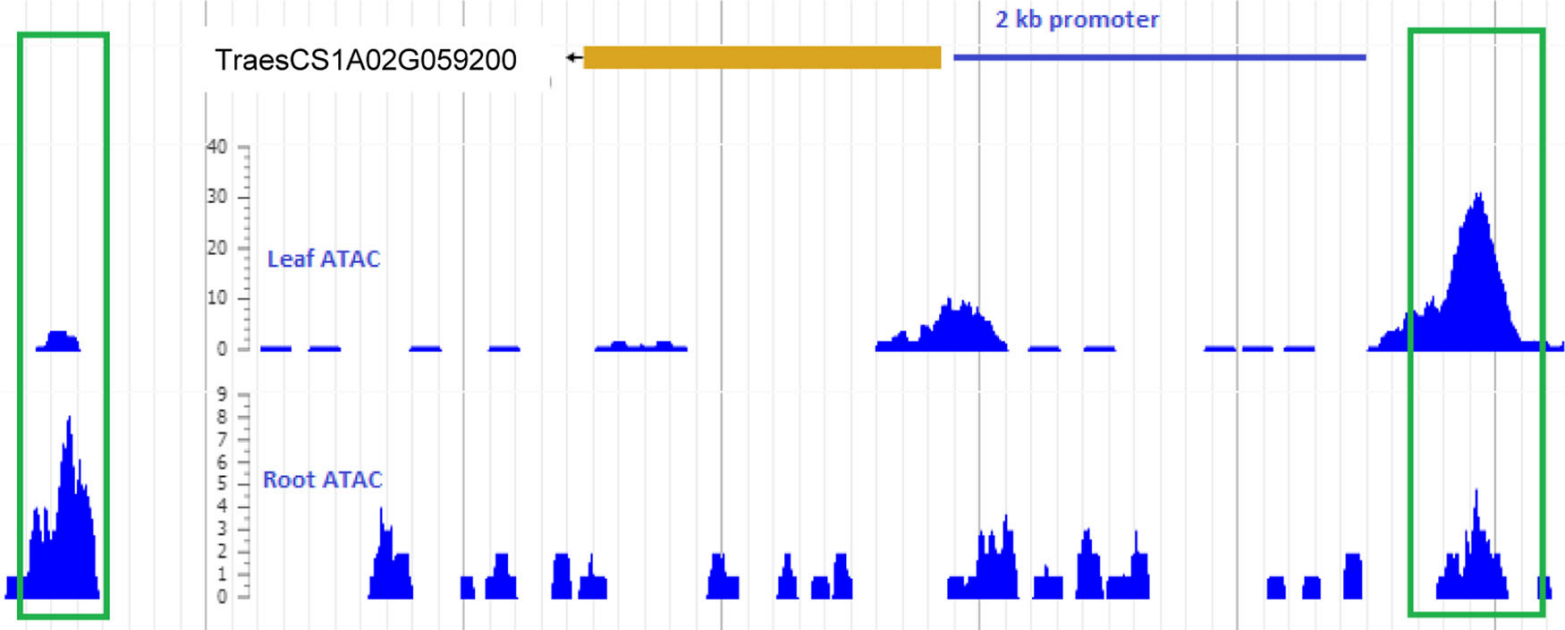
Example of open chromatin regions detected in the leaf protoplast and seminal root tips by assay for transposase‐accessible chromatin (ATAC) using sequencing. The green rectangles indicate open chromatin regions detected outside the 2‐Kbp promoter region

Figure 1 shows an example of a good overlap between peaks from the leaf protoplast and the seminal root tips. It also shows the presence of open chromatin regions located outside the 2‐Kbp regions upstream of the start codons selected for the first promoter capture. The combined analysis of the open chromatin regions revealed that approximately 75% were outside the 2‐Kbp promoter regions (Debernardi et al., 2022), indicating that a large proportion of potential regulatory elements can be missed in captures based only on 2‐Kbp promoter sequences. This emphasizes the importance of complementing promoter regions with open chromatin data to maximize detection of variation in regulatory regions.

The combined promoter and ATAC‐seq data resulted in a target space of 186.0 Mbp. After padding each selected target with 100 nucleotides on either side, the initial sequence used for probe design increased to 241.6 Mbp. After filtering, the final predicted hybridization space was 216.5 Mbp (Supplemental Table S1).

### 3.2 Capture performance

**FIGURE 2:**
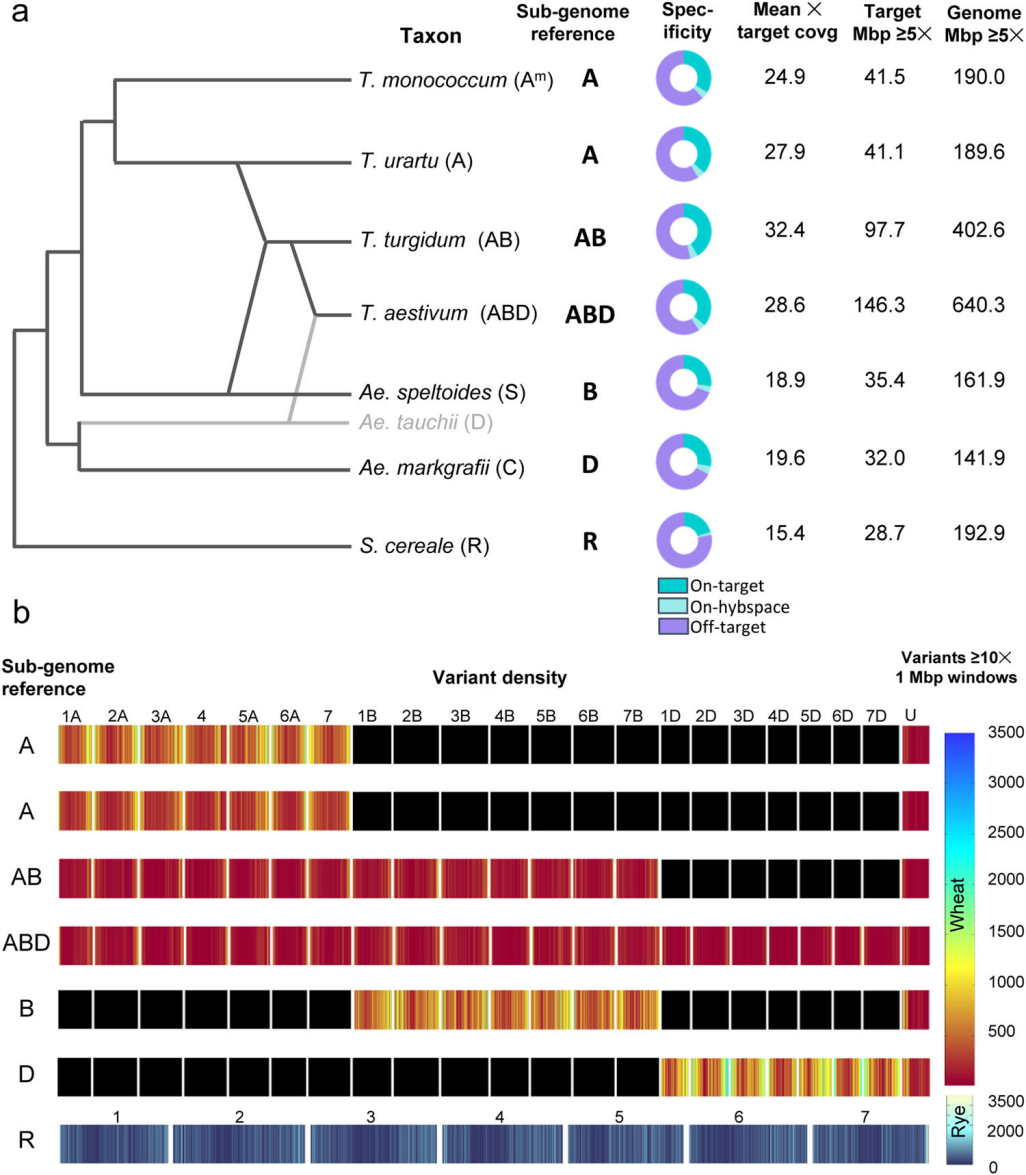
Performance of the new regulatory region capture assay. (a) target coverage depth and genome coverage breadth observed in diploid, tetraploid, and hexaploid wheat accessions and rye. Genome designations are indicated in parenthesis after the species name followed by the subgenomes used as reference for mapping. For diploid libraries, raw read data was down‐sampled to 20‐M read‐pairs, tetraploid to 40 M, and hexaploid to 60 M. Alignment was performed on either the entire Chinse Spring RefSeq v1.0 genome reference, or on the subgenome(s) appropriate for the taxon. Specificity is measured as the proportion of raw reads overlapping the target (blue) or predicted hybridization space (light blue) in the circular graphs. Raw data is presented in Supplemental Tables S2 and S3. Coverage depth and breadth were measured on the target space (∼168 Mbp in hexaploid wheat) and on the entire genome after polymerase chain reaction deduplication and merging of overlapping R1–R2 pairs. (b) Single nucleotide polymorphism (SNP) distribution on different chromosomes; SNPs were called for sites with a minimum quality score of 20 and minimum depth of 10 unique reads

Capture performance was evaluated for specificity (i.e., non‐target exclusion or “reads on‐target”) as well as for coverage breadth and depth. To ensure a fair coverage evaluation between libraries, we sampled identical numbers of read pairs based on sample taxon ploidy, and then aligned the reads to their most closely‐related RefSeq or Weining Rye (sub)genomes (Figure 2a). Our sampling requirements eliminated seven of the 48 sequenced libraries from coverage analysis, but all taxa were represented by at least one library with sufficient data, and in most cases several (Supplemental Table S2).

Performance summary metrics per sample are presented in Supplemental Table S2, and averages per species in Supplemental Table S3 and Figure 2a. Specificity of the assay was measured by counting the proportion of non‐deduplicated reads or read‐pairs that overlapped either the target or hybridization space. Coverage depth and breadth of the target and genome overall were measured following PCR duplicate collapse and consolidation of overlapping R1 and R2 read‐pair coordinates to single intervals. These metrics were calculated using both uniquely mapped reads and reads mapping equally well to more than one genome location. For the latter, the used aligner (*bwa mem*) selects one of the locations at random as the primary alignment. The uniquely mapped reads comprised 79.1 to 79.7% of the reads in tetraploid wheat and 76.4 to 76.9% in hexaploid wheat (Supplemental Table S2).

The average specificity of the eight hexaploid libraries varied depending on the method used from 36.3% on‐target and 40.5% on‐hybridization‐space for single reads, to 39.9% on target and 43.0% on‐hybridization‐space for read‐pairs (Supplemental Table S3). Tetraploid captures were on average more specific (41.7% reads on‐target and 49.8% read‐pairs on‐hybridization‐space). In the diploid species, specificity decreased with increased divergence from the genome used as reference in the assay. *T. urartu*, which is the donor of the A genome in polyploids wheat, showed the highest specificity (36.8% reads on‐target and 46.6% read‐pairs on‐hybridization‐space; Supplemental Table S3), followed by *T. monococcum* (33.7% reads on‐target and 43.2% read‐pairs on‐hybridization‐space) which diverged from *T. urartu* approximately one million years ago (Dubcovsky & Dvorak, 2007). Specificity was further reduced in the more distantly related *Ae. speltoides* (27.1% reads on‐target and 36.3% read‐pairs on‐hybridization‐space) and *Ae. markgrafii* (27.6% reads on‐target and 37.5% read‐pairs on‐hybridization‐space), and the lowest specificity was observed for *S. cereale* genome (20.1% reads on‐target and 27.4% read‐pairs on‐hybridization‐space). In summary, specificity showed a good correlation with the evolutionary history of these species (Figure 2a; Supplemental Table S3).

Per‐base mean coverage of the hybridization‐space (Supplemental Table S3) was highly correlated with assay specificity (*R* = .9871, *P* < .0001). Tetraploid and hexaploid accessions exhibited on average 29.4× and 26.1× hybridization‐space coverage. These values then decreased with phylogenetic distance to 25.4× in *T. urartu*, 22.6× in *T. monococcum*, 18.1 × in *Ae. markgrafii*, 17.2× in *Ae. speltoides*, and 14.4× in rye (Supplemental Table S3). The “Target Region with at Least 5× Coverage” corresponds closely with ploidy level, but within the diploid species it was also affected by specificity and coverage (Supplemental Table S3). This region was 41.1 and 41.5 Mbp in the related diploid species *T. urartu* and *T. monococcum*, respectively, which is approximately half of the target region with at least 5× coverage in tetraploid wheat (97.7 Mbp) and one third of that space in hexaploid wheat (146.3 Mbp). Within the more distantly related diploid species, this space decreased to 35.4 Mbp in *Ae. speltoides*, 32.0 Mbp in *Ae. markgrafii*, and 28.7 Mbp in rye (Supplemental Table S3).

We also checked the number of promoters from high‐confidence annotated genes that were intersected by the sequencing space with at least 5× coverage (Supplemental Table S4). This intersection included on average 96.8% of the genes in hexaploid wheat, 93.5% in tetraploid wheat, and 85.7 to 91.8% in *T. urartu*, *T. monococcum*, *Ae. speltoides*, and *Ae. markgrafii*. Genes located in the unknown chromosome (chrUn) outside of the compared genomes likely contributes to the lower proportion of intersected genes in tetraploid and diploid species. In summary, this assay captures a significant portion of the promoters of most tested species.

Although the target and hybridization space experienced the highest enrichment, some regions outside of this space achieved moderate coverage consistently across samples within the same taxon. Of these moderately covered, non‐hybridization‐space regions, 38 to 60% were shared among all samples of the same taxon, suggesting a non‐random distribution of the off‐target reads across the non‐targeted space. These shared regions may include sequences similar to those targeted but not included in the assay, residual repeats, or be the result of amplification bias. Regardless of its origin, the additional consistent moderate‐coverage genome space delivered by the assay may be useful for general‐purpose variant discovery or genotyping.

Called variant densities are depicted in Figure 2b for one representative sample for each taxon and for sites with a minimum of 10 × unique read coverage. The SNP densities and distribution are broadly consistent with the genome biology and known level of sequence divergence between the different tested taxa and the hexaploid reference genome. For instance, the A^m^ genome from *T. monococcum* and the B‐like genome of *Ae. speltoides* show the highest density of variants and the D genome of hexaploid wheat shows the lowest. In the wheat genome, genes and their regulatory elements are concentrated in the distal regions, and this is recapitulated in the variant density plots. The lower frequency of variants close to the centromeric regions is evident in several of the chromosomes.

Rye is an outcrossing species with high levels of polymorphism, which is also reflected in the distribution of variants along its seven chromosomes. This high level of polymorphism may help explain the highly divergent performance metrics of the two rye specimens analyzed here, both in terms of read mappability to the genome, as well as specificity. In contrast, for the wheat species, overall performance was highly consistent both among different representatives of the same taxon, and among replicate libraries from the same genomic DNA source.

In summary, this capture assay was very efficient to capture variation in the regulatory regions of wheat and its closely related species but showed a lower specificity and covered a smaller target space when used in more distantly related species such as rye.

### 3.3 Comparisons of regulatory capture assays

The same 24 tetraploid wheat genomic DNA samples that were library‐prepped and captured with this new regulatory design were also separately library‐prepped and captured using a previous promoters‐specific probe design (Gardiner et al., 2019) and the SeqCap EZ platform (Roche). To make an even comparison, we down‐sampled to 40‐M read‐pairs (80‐M reads) per library from both experiments. We mapped the reads to the Chinese Spring RefSeq v1.0 (A, B and unknown genome plus Kronos sequences) and identified EMS mutations using the MAPS pipeline (see Materials and Methods). We discovered an average of 3,338.6 and 3,131.5 EMS mutations per line with the Arbor and Gardiner probe sets and protocols, respectively (6.6% increase, *P* < .001; Table 1; Supplemental Table S5). Both captures showed good proportion of mapped reads and similar proportion of G to A or C to T mutations, which are typically generated by EMS.

**TABLE 1:** Comparison between the Gardiner probe set, and new wheat regulatory capture described in this study. Data are presented as the mean statistics of all 24 analyzed libraries

Trait	Arbor	Gardiner probe set	% Improvement Arbor / Gardiner
Raw read‐pairs analyzed, No.	40,000,000	40,000,000	same
% Reads mapped uniquely	79.4	71.6	10.9***
% Reads on target	41.7	28.5	46.3***
% Read‐pairs on target	47.0	29.3	60.4***
Coverage depth in target region^a^	32.37 ×	7.56 ×	328***
CT or GA mutations detected per library^b^, No.	3,338.6	3,131.5	6.6***
% CT or GA mutations^b^	95.6	94.7	0.9*

^a^ Either the ∼114.3 Mbp across subgenomes A, B, and Un in DAB_WheatRegulatoryV1.IWGSCv1.bed, or the ∼227.3 Mbp across A, B and Un in Prom‐capture‐HC+5UTR‐targets.bed from Gardiner et al., 2019.

^b^ Ethyl methanesulfonate (EMS) mutations result in CT or GA single nucleotide polymorphisms (Supplemental Table S5).

* Significant at the .05 probability level.

*** Significant at the .001 probability level.

Compared with the Gardiner probe set and protocol, the Arbor assay and protocol showed a significant (*P* < .001) increase in specificity (46.3 and 60.4% increases in reads and read‐pairs on target, respectively) and coverage in the target region (3.3‐fold increase; Table 1). The total number of reads mapping to unique positions in the genome was 10.9% higher for the Arbor system, which indicates a higher rate of reads derived from putative single‐copy loci. This particular metric should be robust to variation in library preparation between protocols, though differences in pre‐ and post‐capture PCR amplification could contribute to the differences.

Taken together, these results suggest that the more stringent filtering of repetitive regions may have contributed to a significant reduction in duplicated reads and an increase of reads on target in the new protocol relative to the original one, although it is also possible that the different protocols contributed to these differences. Regardless of the cause of the differences, the new design and capture system described here represents an improvement in overall performance compared with our trials of the previous capture product.

## 4 DISCUSSION

The same myBaits technology used for this new regulatory capture design was used successfully previously in a more limited analysis of a subset of wheat gene promoters (Hammond‐Kosack et al., 2021). That study explored the 1.7‐Kbp upstream of the coding regions of 459 wheat genes associated with agriculturally important traits in 95 ancestral and commercial wheat accessions. The study revealed a high level of conservation in the wheat promoter regions but also discovered many SNPs and indels located within predicted plant transcription factor binding sites. The new myBaits assay developed here expands the analyzed promoter regions by 200‐fold to the promoters of all high confidence genes in the CS RefSeq v1.0 annotation.

A key special feature of this new capture assay is that it targets additional 23.5 Mbp of open chromatin detected with leaf and root ATAC‐seq data (Debernardi et al., 2022; Lu et al., 2020) outside of the 2‐Kbp promoter regions. Because transcription factors require open chromatin to exert their regulatory functions, the identified ATAC‐seq regions present an excellent tool for identifying potential regulatory regions in the genome. Our analysis of the root ATAC‐seq data showed a distribution of peaks among genic, promoter and intergenic regions that was similar to ATAC‐seq data reported from other plant species (Maher et al., 2018). This open chromatin distribution indicates that a large proportion of putative regulatory regions can be missed by focusing only in the 2‐Kbp regions upstream the start codons.

We observed a significant overlap (60%) between the ATAC‐seq peaks detected in the leaf protoplast and the seminal root tips, in spite of the divergent nature of the tissues and conditions investigated. This level of overlap is comparable with the 71% overlap detected between ATAC‐seq data from the more related root hairs and non‐hair root cells reported in Arabidopsis (Maher et al., 2018). In addition, more than 99% of the peaks detected in the root frozen tissues were validated in an independent ATAC‐seq study using fresh root tissues that yielded seven‐fold more peaks (Debernardi et al., 2022). The presence of a substantial number of tissue specific peaks (∼40%) suggests that a more complete inventory of open chromatin regions will require additional ATAC‐seq data from different wheat tissues, developmental stages and stress conditions. Furthermore, multiple complete wheat genomes are now available, which can contribute new or more divergent regulatory regions. As these sites are accrued and curated, they can be added as patches or separate modules to the core assay designed here.

Ultimately, targeted sequencing assays like this one are meant to reduce the cost of sequencing regions of interest. Though exact costs will vary with ploidy, divergence from the reference, and number of samples, the assay would typically reduce materials and sequencing costs by 70–80% per sample compared with 10× whole genome Illumina sequencing. In addition, the probe design itself and the myBaits platform more generally are compatible with long‐insert libraries for eventual sequencing on PacBio or Oxford Nanopore sequencing platforms, which may result in even larger cost reductions compared with long‐read, whole‐genome sequencing. This technique is already well‐established for targeted sequencing of large (3 Kbp and longer) genomic and transcriptomic material, especially for characterizing nucleotide‐binding site (NBS)‐leucine‐rich repeat (LRR) resistance genes (Giolai et al., 2016; Seong et al., 2020; Witek et al., 2016).

In summary, this capture assay represents a versatile and cost‐effective tool for targeted sequencing of regulatory region of the wheat genome and of related Triticeae species that can be adapted to different sequencing platforms. We anticipate that data retrieved by this capture assay can be used to characterize the extent of natural variation in regulatory regions across diverse Triticeae species and to detect conserved binding sites of transcription factors. This assay will also be a useful discovery tool for identifying regulatory variants associated with phenotypic variation, which can be then incorporated into larger‐scale genotyping platforms for wheat breeding programs.

## AUTHOR CONTRIBUTIONS

Junli Zhang: Data curation; Formal analysis; Investigation; Software; Validation; Visualization; Writing – original draft. Juan M. Debernardi: Investigation; Resources; Writing – review & editing. Germán  F. Burguener: Formal analysis; Software; Writing – review & editing. Frédéric Choulet: Formal analysis; Software; Writing – review & editing. Etienne Paux: Formal analysis; Software; Writing – review & editing. Lauren O'Connor: Formal analysis; Software; Writing – review & editing. Lauren O'Connor: Data curation; Investigation; Writing – review & editing. Jacob Enk: Conceptualization; Data curation; Formal analysis; Funding acquisition; Methodology; Project administration; Resources; Software; Visualization; Writing – review & editing. Jorge Dubcovsky: Conceptualization; Data curation; Funding acquisition; Investigation; Project administration; Supervision; Visualization; Writing – review & editing

## CONFLICT OF INTEREST

Jacob Enk is employed by Daicel Arbor Biosciences, which sells this new assay. Lauren O'Connor is a former employee of Daicel Arbor Biosciences. The other authors declare that they do not have any conflicts of interest.

## Supporting information


**Table S1**. Target and hybridization space of the new promoter capture by genome.
**Table S2**. Capture specificity and coverage in the new capture and the captures with the probe set from Gardiner et al. 2019
**Table S3**. Summary data used for Figure 2 (mean values of Table S2 per taxon)
**Table S4**. Number of intersected genes with the sequencing spaces with at least 5× coverage.
**Table S5**. Number of mutations in 19 libries from Kronos EMS mutants using the Abor andGardiner captures and protocols.

## Data Availability

Data is publicly available and deposited in Zenodo (Zhang et al., 2022). The files include: browser extensible data (BED) files for Target space wheat (DAB_WheatRegulatoryV1.IWGSCv1.bed.gz), hybridization space wheat (DAB_WheatRegulatoryV1.IWGSCv1_hybspace.bed.gz), Target space rye (DAB_WheatRegulatoryV1.Weiningv1.bed.gz), and hybridization space rye (DAB_WheatRegulatoryV1.Weiningv1_hybspace.bed.gz), the FASTA file for wheat predicted hybridization space (DAB_WheatRegulatoryV1.IWGSCv1_hybspace.fa.gz), 5× coverage space for each species, and all the Building information modelling (BIM) collaboration format (BCF) files for variant callings used in Figure 2b. Probe sequences (DAB_WheatRegulatoryV1_probes.fas.gz) can be downloaded from the same web site and from Daicel Arbor Biosciences. The BioProject numbers are PRJNA894226 (*Triticum aestivum*), PRJNA894228 (*Triticum turgidum*), PRJNA895032 (*Triticum monococcum*), PRJNA895087 (*Triticum urartu*), PRJNA895093 (*Secale cereale*) and PRJNA895045 (*Aegilops markgrafii* and *Aegilops speltoides*). All Kronos mutant lines are available upon request from University of California‐Davis and from the John Innes Center in the UK. The capture Assay is commercially available from Arbor Biosciences.
